# Nutritional Composition and Safety Aspects of Deep-Sea Whelks (*Buccinum tenuissimum* Kuroda)

**DOI:** 10.3390/foods13081169

**Published:** 2024-04-11

**Authors:** Sana Mansoor, Jin-Hwa Lee, Khawaja Muhammad Imran Bashir, Jae-Hak Sohn, Jae-Suk Choi

**Affiliations:** 1 Department of Seafood Science and Technology, Institute of Marine Industry, Gyeongsang National University, Tongyeong 53064, Republic of Korea; sanamansoorahmad@gmail.com (S.M.); evolution_5237@gnu.ac.kr (J.-H.L.); imranbashir@gnu.ac.kr (K.M.I.B.); 2 German Engineering Research and Development Center for Life Science Technologies in Medicine and Environment, Busan 46742, Republic of Korea; 3 Department of Food Science and Culinary Arts, College of Health and Welfare, Silla University, Busan 46958, Republic of Korea

**Keywords:** Buccinidae, deep-sea whelk, fatty acids, heavy metals, proximate composition, safety, seafood

## Abstract

The deep-sea whelk *Buccinum tenuissimum* Kuroda is highly sought-after as food in East Asian countries, notably, Korea and Japan. However, it lacks official recognition as a food product in Korea. This study aimed to assess its nutritional composition and safety for the potential development of seafood products. The nutritional analysis revealed high protein (13.54–20.47 g/100 g whelk), fat (0.85–8.59 g/100 g whelk), carbohydrate (1.55–12.81 g/100 g whelk), and dietary fiber (1.25–1.95 g/100 g whelk) contents in both muscle and gut samples, with energy contents ranging from 339.11 ± 1.64 to 692.00 ± 3.21 kJ/100 g. Key minerals, including iron, potassium, calcium, and sodium, and essential fatty acids, including eicosapentaenoic acid, docosahexaenoic acid, arachidonic acid, omega-3, and omega-6 fatty acids, were abundant, making it a potential supplementary food. Notably, heavy metal levels met the Korean standards for seafood safety. No trans fats, radioactivity concerning the radioactive isotopes ^134^Cs/^137^Cs and ^131^I, or pathogenic bacteria were detected. This confirms the safety and nutritional value of deep-sea whelks, suggesting their potential for developing seafood products rich in beneficial components, which could enhance nutrition and food security while contributing to economic growth.

## 1. Introduction

The deep sea, a captivating, mysterious realm, harbors an ecosystem brimming with unique marine species, many of which remain unexplored [1,2]. Among these inhabitants, the whelk species *Buccinum tenuissimum* Kuroda emerges as a compelling subject of study, providing insights into both the ecological intricacies of the deep sea and its potential as a nutritional resource [3,4]. With their distinct morphology and adaptation to extreme environmental conditions, deep-sea whelks offer an intriguing avenue for understanding the dynamics of life in the deep ocean [1,5].

Countries like Korea and Japan are notable consumers of whelks worldwide. However, species such as moon snails (*Glossaulax didyma*) and finely-striated buccinum (*B*. *striatissimum*) face overfishing due to their shallow-water habitats. Meanwhile, the deep-sea whelk *B. tenuissimum* (known as “큰물레고둥—Keunmullegodung” in Korean), belonging to the gastropod family Buccinidae and found in the depths (400 to 1000 m) of the East Sea [6], presents unique characteristics, including its thin and brittle shell measuring approximately 11–17 cm and a distinct yellow-green or black coloration [7,8]. Despite its popularity in Korean cuisine, comprehensive studies on its nutritional composition and safety aspects are notably lacking, highlighting a gap in understanding regarding its potential as a valuable food resource.

Similar to trends observed in Nigeria [3], whelks are gaining popularity in Korean communities and are often consumed with rice or soups as alternatives to beef or fish. Despite being a highly relished delicacy, the nutritional potential of whelk meat remains poorly documented. To address this gap, we identified *B. tenuissimum* as a potential resource for domestic whelk production, particularly off Ulleungdo Island’s coast. This giant deep-sea whelk boasts relatively abundant resources, larger muscle size, and a darker shell compared to the spinning whelk, offering a more delicious and flavorful experience [8]. However, obstacles exist due to its lack of registration as a food material [9], hindering local production.

This research aims to unveil the nutritional composition of *B. tenuissimum*, shedding light on its biological intricacies and significance for human consumption. By scrutinizing its macro- and micronutrient contents [10] and addressing safety concerns, such as heavy metal accumulation, microbial contamination, and other factors critical to food safety [11,12], we seek to contribute to marine biology, nutrition, and food safety fields. Ultimately, this study may facilitate sustainable exploitation and utilization of deep-sea whelk resources, potentially advancing marine-based nutrition and aquaculture practices.

## 2. Materials and Methods

### 2.1. Samples

**Figure 1:**
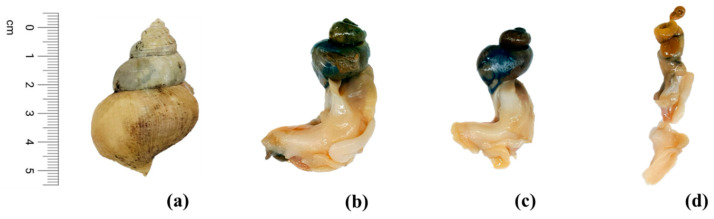
Deep-sea whelks (*B. tenuissimum* Kuroda) used in this study. (**a**) Outer shell of whelk. (**b**) Large-sized whelk. (**c**) Medium-sized whelk. (**d**) Small-sized whelk.

Frozen deep-sea whelks (*Buccinum tenuissimum*), harvested in the East Sea of Korea in January 2023, were kindly provided by BaekHwa Co., Ltd., Busan, Republic of Korea. The whelks were categorized into large-sized, medium-sized, and small-sized groups, with both muscle and gut samples collected from each group for this study. Measurements were taken with a digital caliper (5100-IP54; Wenzhou Sanhe Measuring Instrument Co., Ltd., Wenzhou, China). After dissection (see Figure 1), the weights of different body parts, such as shells, muscles, and guts, were determined using an analytical balance (CP224S Balance; SATORIUS, Göttingen, Germany).

In this study, 100 g muscle and 100 g gut samples were used. Approximately, 5–6 large-sized whelks yielded the required 100 g for muscle and gut samples, whereas 10–12 medium-sized whelks and 20–22 small-sized whelks were required to obtain samples of the same weight. Additionally, whelks with intact shells and a good appearance were selected for inclusion in this study.

### 2.2. Proximate Composition Analysis

The analysis of the proximate composition, including moisture, crude protein, crude fat, ash, and total carbohydrates, was performed following the methods listed by the Association of Official Analytical Chemists (AOAC) [13]. Briefly, moisture was measured by atmospheric heating and drying, crude protein by the micro-Kjeldahl method, crude fat by the Soxhlet method, and ash by the ash method, all as described by the AOAC [13]. Total carbohydrates were calculated following the method described in the Korean Food and Drug Administration (KFDA) food code [14]. Energy values were calculated using the Atwater energy conversion factor for crude protein, crude fat, and total carbohydrates [13].

#### 2.2.1. Mineral Content Analysis

The mineral content was analyzed by preparing samples through wet decomposition following the Standard Food Test Method described by the KFDA [14]. Briefly, 5.0 g of each sample was mixed with 10 mL of 6 M nitric acid (HNO_3_) in a 250 mL decomposition flask, heated, and dried. Quantities of 10 mL of nitric acid solution (HNO_3_:H_2_O = 1:2) and 10 mL of 0.6 M perchloric acid (HClO_4_) solution were added to the dried content. When it became colorless, it was heated at 100 °C for 30 min. The colorless solution was diluted with 20 mL of distilled water, transferred to an evaporation dish, and heated again at 100 °C to evaporate HClO_4_. Afterwards, it was mixed with 10 mL of 3.78 M hydrochloric acid solution (HCl:H_2_O = 1:2), dissolved completely in a water bath at 80 °C, and the final volume was adjusted to 100 mL. The prepared solution was used as a sample for mineral analysis. An atomic absorption spectrophotometer (AAS-3300; PerkinElmer Inc., Waltham, MA, USA) was used to measure calcium (Ca), potassium (K), and iron (Fe) at 422 nm, 589 nm, and 248 nm, respectively, while phosphorus (P) was measured with a spectrophotometer (UV-2450; Shimadzu Co., Ltd., Kyoto, Japan) at 470 nm, according to the molybdenum blue colorimetric method [14]. The mineral contents were quantified after comparison with the standard solutions for Ca (21049; Sigma-Aldrich, St. Louis, MO, USA), K (96665; Sigma-Aldrich), Fe (16596; Sigma-Aldrich), and P (P3869; Sigma-Aldrich).

#### 2.2.2. Measurement of Dietary Fiber Content

Dietary fiber content was measured according to the Food Standards Method described by the KFDA [14]. The dried samples were continuously decomposed with α-amylase, protease, and amyloglucosidase enzymes to remove carbohydrates and proteins. The mixtures were then precipitated with ethanol, filtered, washed with ethanol and acetone, and dried, and the weights were measured.

#### 2.2.3. Quantification of Free Sugars

Ten grams of each sample was mixed with 100 mL of distilled water, and the free sugars were extracted using a reflux condenser at 70 °C for 40 min, following the method described by the Korean Ministry of Food and Drug Safety (MFDS) [15]. The extract was filtered and then concentrated at 40 °C using a vacuum concentrator (EYELA rotary evaporator N-1200; Tokyo Rikakikai Co., Ltd., Tokyo, Japan). The concentrate was transferred to a separatory funnel, mixed with 20 mL of hexane, and left to stand still to remove fat-soluble substances. The obtained residue was used to quantify the free sugar content by High-Performance Liquid Chromatography (HPLC) using a Waters Isocratic 510 pump (Waters Corporation, Milford, MA, USA) with a SpectraSYSTEM RI-150 detector (Thermo Fisher Scientific, Rockford, IL, USA), equipped with a YMC-Pack Polyamine II column (4.6 × 250 mm; YMC Co., Ltd., Kyoto, Japan). The mobile phase consisted of 75% acetonitrile and 25% HPLC-grade water. The flow rate was adjusted to 1.0 mL/min with an injection volume of 20 μL.

#### 2.2.4. Quantification of Cholesterol

Referring to the cholesterol extraction method in the General Test Methods of the Food Codex [15], 5 g (±0.05) of each sample was added to an extraction tube containing 6% pyrrogallol (in ethanol), vortexed for 2 min, and sonicated for 10 min. Later, 8 mL of 60% potassium hydroxide (KOH) solution was added to the extraction tube and vortexed for 2 min, and the air in the tube air was replaced with nitrogen gas (N_2_). The mixture was allowed to react at 75 °C for 60 min in a shaking water bath at 100 rpm, cooled to room temperature, and mixed with 20 mL of 2% sodium chloride (NaCl) solution. After adding 15 mL of extraction solvent [n-hexane:ethyl acetate = 85:15 (*v*/*v*) and 0.01% butylated hydroxy toluene] and overtaxing for 2 min, the supernatant was separated and passed through a sodium sulfate column (repeated 3 times). The supernatant was transferred to a 50 mL volumetric flask, and after removing 12.5 mL of the extract solvent with N_2_ gas, it was re-dissolved in 3 mL dimethylformanide (DMF). To derivate cholesterol, 2 mL of hexamethyldisilane and 0.1 mL of trimethylchlorosilane were mixed and allowed to react at room temperature for 15 min. Later, 1 mL of an internal standard solution (0.1 mg/mL 5-α-cholestane in heptane; c8003; Sigma-Aldrich) and 10 mL of distilled water were added and stirred, and the supernatant was separated by centrifugation (3000× *g* for 2 min) to remove moisture.

Cholesterol was then quantified with a gas chromatography–flame ionization detector (GC-FID; HP 6890 Plus; Hewlett Packard Ltd., Palo Alto, CA, USA) equipped with an HP-5 column (25 m × 0.32 mm × 0.17 μm; Agilent Technologies Inc., Santa Clara, CA, USA). The flow rate was adjusted to 2.0 mL/min, and helium was used as the carrier gas. The temperature of the injector and the detector was set at 250 °C and 300 °C, respectively. The temperature of the oven was maintained at 190 °C for 2 min, raised to 230 °C at 20 °C per min and maintained for 3 min, and heated to 270 °C at 40 °C per min and maintained for 25 min. A cholesterol standard solution (5-α-cholestane, Sigma-Aldrich) was used to obtain a standard curve, and the cholesterol content was expressed as mg/100 g of the sample.

#### 2.2.5. Vitamin D_3_ Content Analysis

One gram of each sample was mixed with 30 mL of 95% methanol and sonicated at 60 °C for 40 min. The volume of the sample mixture was adjusted to 50 mL with 95% methanol and filtered through a 0.45 μm nylon syringe filter (Merck KGaA, Darmstadt, Germany). HPLC analysis of the vitamin D_3_ content was performed using a Waters Isocratic 510 pump (Waters Corporation) with a UV/Vis detector (440 nm and 570 nm), coupled to a CAPCELL PAK C18 column (4.6 × 250 mm; Osaka-Soda Co., Ltd., Tokyo, Japan). The mobile phase consisted of 95% methanol and 5% HPLC-grade water. The flow rate was adjusted to 1.0 mL/min. Each sample was injected at a volume of 20 μL and detected at a wavelength of 256 nm.

### 2.3. Amino Acid Analysis

The amino acids were separated by HPLC using a Waters Isocratic 510 pump (Waters Corporation) with a SpectraSYSTEM RI-150 detector (Thermo Fisher Scientific), equipped with a YMC-Pack Polyamine II column (4.6 × 250 mm; YMC). Each sample was injected at a volume of 20 μL. The mobile phase consisted of 75% acetonitrile and 25% HPLC-grade water. The flow rate was adjusted to 1.0 mL/min.

The HPLC-separated amino acids were analyzed using an amino acid auto analyzer (L-8900; Hitachi Ltd., Tokyo, Japan) with a UV/Vis detector (440 nm and 570 nm), coupled to an ion exchange column (2622PH column; 4.6 × 60 mm, Hitachi High-Technologies Corporation, Tokyo, Japan). Each sample was injected at a volume of 20 μL. A buffer set (PH-SET; Kanto Chemical Co., Inc., Tokyo, Japan) was used as a mobile phase, and the flow rates of ninhydrin and buffer were adjusted to 0.35 mL/min and 0.40 mL/min, respectively.

#### 2.3.1. Bound Amino Acid Analysis

The bound amino acids were analyzed by the ninhydrin post-column reaction method described by the AOAC [13] using ion-exchange chromatography. Two hundred micrograms of each sample and 10 mL of 6 M HCl were added to a decomposition tube, injected with N_2_ gas, hydrolyzed at 110 °C for 24 h, and filtered using a cellulose acetate syringe filter (Corning syringe filters, Merck KGaA, Darmstadt, Germany). After concentrating the filtrate with a vacuum concentrator (N-1110; EYELA, Tokyo, Japan), the volume was adjusted to 50 mL with 0.2 M sodium citrate buffer and filtered again with a 0.45 μm nylon syringe filter (Corning, Corning, NY, USA). To determine sulfur-containing methionine and cysteine amino acids, performic acid oxidation [16] was used, and for tryptophan, the alkaline hydrolysis method [17] was used.

#### 2.3.2. Free Amino Acid Analysis

For free amino acid analysis, 2 g of each sample was mixed with 15 mL of 0.02 M HCl, vortexed for 1 min, and allowed to react at room temperature for 1 h, as described in the methods for free amino acid analysis [15]. The volume of the sample mixture was adjusted to 50 mL with 0.02 M HCl and centrifuged at 10,000× *g* for 10 min. The supernatant was carefully removed and mixed with 10 mL of 5% trichloroacetic acid (TCA) per 5 mL supernatant, vortexed for 1 min, and centrifuged at 3000× *g* for 10 min. Then, 5 mL of the supernatant was separated, mixed with 5 mL of n-hexane, and centrifuged again at 3000× *g* for 10 min. After centrifugation, the supernatant was separated, filtered through a 0.20 μm cellulose acetate syringe filter (Corning), and used as a sample for free amino acid analysis.

### 2.4. Fatty Acid Composition Analysis

For the fatty acid composition analysis, 10 g of each sample was mixed with 100 mL of a chloroform and methanol (2:1) solution, extracted at room temperature for 24 h, and then concentrated under reduced pressure, following the method described by Folch et al. [18]. After methyl esterification of the extracted lipids with a 14% boron trifluoride–methanol (BF_3_-MeOH; Sigma-Aldrich) solution, they were analyzed with a GC-FID (Hewlett Packard) coupled to an HP-88 column (100 m × 0.25 mm, 0.2 μm; Agilent Technologies). The column temperature was maintained at 140 °C for 5 min, heated at 4 °C per min, and maintained at 240 °C for 20 min. The inlet temperature was maintained at 260 °C, and the Agilent Flame Ionization Detector (FID; HP 6890 Plus; Agilent Technologies) was kept at 270 °C. N_2_ gas was used as the carrier gas at a flow rate of 1 mL/min, and the split ratio was adjusted to 1/50. A sample volume of 1 μL was injected, and the fatty acid peaks were confirmed by comparing the retention times of the methyl esters with those of a standard 37-component fatty acid methyl ester (FAME) mix (CRM47885; Supelco, St. Louis, MO, USA). The analyzed individual fatty acid contents were obtained by calculating the area ratio of each fatty acid to the total fatty acid area and expressed as a percentage of each fatty acid.

### 2.5. Lipid Nutritional Quality Indices

Besides classical indices, such as total saturated fatty acids (ΣSAFAs), total monounsaturated fatty acids (ΣMUFAs), total polyunsaturated fatty acids (ΣPUFAs), total omega-3 fatty acids (Σω3), total omega-6 fatty acids (Σω6), and the ratio of omega-6 to omega-3 fatty acids (ω6/ω3; *n*-6/*n*-3 ratio), we also calculated other nutritional quality indices. These included the atherogenic index (AI), the thrombogenic index (TI), and the hypocholesterolemic/hypercholesterolemic index (h/H) as described by Ulbricht and Southgate [19] and Mierliță [20], using the following empirical equations (Equations (1)–(3)):

AI = [C12:0 + (4 × C14:0) + C16:0]/∑UFA(1)

TI = [C14:0 + C16:0 + C18:0]/[(0.5 × ∑MUFA) + (0.5 × ∑*n*-6 PUFA) + (3 × ∑*n*-3 PUFA) + (∑*n*-3 PUFA/∑*n*-6 PUFA)] (2)

h/H = (cis − C18:1 + ∑PUFA)/(C12:0 + C14:0 + C16:0)(3)

where UFA stands for unsaturated fatty acid, MUFA stands for monounsaturated fatty acid, and PUFA stands for polyunsaturated fatty acid.

### 2.6. Heavy Metal Analysis

For lead (Pb), cadmium (Cd), and arsenic (As), the samples were pre-treated according to the Food and Drug Administration test method [15] using a microwave (QWAVE 2000; Questron technologies Corp., Mississauga, ON, Canada). The samples were then analyzed using an inductively coupled plasma (ICP) spectrometer (IRIS Intrepid II XDL ICP-OES, Thermo Fisher Scientific). The analytical conditions of the ICP spectrometer used for metal analysis are shown in Table S1. Furthermore, mercury (Hg) analysis was directly performed using a gold amalgam analyzer (AMA 254; Leco Inc., St. Joseph, MI, USA).

### 2.7. Radioactivity Analysis

The radioactivity of the samples was analyzed for radioactive isotopes, such as ^134^Cs/^137^Cs and ^131^I, using standard electrode coaxial Ge detectors (Mirion Technologies, Canberra, Inc., Atlanta, GA, USA). The experiment was performed at the Busan National University Radioactivity Analysis Center, Busan, Republic of Korea, following the method described by the MFDS [21].

### 2.8. Microbial Analysis

Total bacterial counts (TBCs), *Escherichia coli*, and coliforms in the whelk samples were examined according to the methods described by the MFDS [15]. The samples were homogenized in sterile bags containing sterile saline at a ratio of 1:9 (*w*/*v*) using a Stomacher 400 Circulator (Seward Ltd., West Sussex, UK) for 3 min. Difco plate count agar (BD Co., Franklin Lakes, NJ, USA) and EC medium (BD Co., Franklin Lakes) were used to measure the TBCs, *E. coli*, and coliforms. The plates were incubated at 35 °C for two days in an incubator (SIR-20; SciLab Co., Ltd., Seoul, Republic of Korea), and bacterial counts were recorded.

### 2.9. Statistical Analyses

All experiments were performed in three replications, each containing 25 whelks. The results are expressed as means ± standard deviations (SDs). One-way analysis of variance (ANOVA) and the Student’s *t*-test were employed using SPSS Statistics version 24.0 (SPSS Inc., Chicago, IL, USA), and the experimental results were considered statistically significant at *p* < 0.05.

## 3. Results

### 3.1. Physical Characteristics

Frozen deep-sea whelks were categorized based on their average shell height (H) × breadth (W): large-sized whelks had an H × W greater than 73.23 × 40.98 mm, medium-sized whelks had an H × W greater than 54.07 × 29.17 mm, and small-sized whelks had an H × W greater than 40.43 × 25.38 mm. The corresponding weights for large-, medium-, and small-sized whelks were 55.90 ± 5.97, 31.88 ± 8.63, and 14.41 ± 2.42 g, respectively. The shell, muscle, and gut weights were also measured, with shell weights at 9.06 ± 2.09, 6.69 ± 1.70, and 3.87 ± 0.74 g; muscle weights at 20.18 ± 3.68, 12.42 ± 3.68, and 5.51 ± 1.40 g; and gut weights at 23.94 ± 4.17, 11.66 ± 5.59, and 3.74 ± 0.91 g, respectively (Table 1).

### 3.2. Proximate Composition

Proximate composition analysis revealed varying levels of crude protein, carbohydrate, sugars, fat, saturated fat, crude ash, K, and dietary fiber in whelks, ranging from 13.54 ± 0.04 to 20.47 ± 0.08, 1.55 ± 0.02 to 12.81 ± 0.04, 0.80 ± 0.004 to 1.40 ± 0.03, 0.85 ± 0.01 to 8.59 ± 0.01, 0.32 ± 0.01 to 2.14 ± 0.05, 2.15 ± 0.03 to 2.81 ± 001, 0.35 ± 0.002 to 0.38 ± 0.001, and 1.25 ± 0.006 to 1.95 ± 0.009 g/100 g, respectively. Cholesterol, Na, Ca, and Fe contents ranged from 110.30 ± 0.54 to 469.57 ± 2.41, 482.69 ± 2.45 to 715.87 ± 3.62, 57.72 ± 0.30 to 284.58 ± 1.53, and 0.87 ± 0.007 to 6.09 ± 0.04 mg/100 g, respectively. Calorie contents varied from 339.11 to 692.00 kJ/100 g. Meanwhile, trans fats and vitamin D_3_ were not detected in the studied whelks (Table 2).

### 3.3. Amino Acid Composition

The bound amino acid composition of the studied whelks included aspartic acid (1.21–1.89%), threonine (0.54–0.87%), serine (0.53–0.72%), glutamic acid (1.80–2.42%), proline (0.53–0.73%), glycine (0.76–0.93%), alanine (0.75–1.00%), valine (0.52–0.98%), isoleucine (0.40–0.86%), leucine (0.88–1.45%), tyrosine (0.29–0.47%), phenylalanine (0.40–0.84%), histidine (0.25–0.51%), lysine (0.80–1.41%), arginine (0.94–1.15%), cysteine (0.20–0.54%), methionine (0.33–0.57%), and tryptophan (0.07–0.83%). The muscles and guts also showed various free amino acids, including taurine (1251.29–1697.33 mg/kg), aspartic acid (473.23–619.50 mg/kg), threonine (222.60–298.78 mg/kg), serine (293.67–373.93 mg/kg), glutamic acid (662.21–1017.92 mg/kg), sarcosine (1025.02–1663.61 mg/kg), hydroxy proline (35.84–130.45 mg/kg), proline (307.14–475.03 mg/kg), glycine (338.34–427.26 mg/kg), alanine (584.17–661.01 mg/kg), valine (269.31–371.37 mg/kg), methionine (143.15–194.64 mg/kg), isoleucine (177.34–284.03 mg/kg), leucine (383.72–575.09 mg/kg), tyrosine (228.32–336.61 mg/kg), phenylalanine (157.29–277.88 mg/kg), γ-amino-n-butyric acid (2.07–2.89 mg/kg), ethanol amine (26.25–157.47 mg/kg), ammonia (86.16–170.26 mg/kg), ornithine (23.75–102.96 mg/kg), lysine (299.65–537.61 mg/kg), histidine (143.15–219.05 mg/kg), and arginine (737.86–1400.78 mg/kg). The free amino acids had a total value ranging from 8188.32 ± 29.82 mg/kg to 10,680.9 ± 37.93 mg/kg whelk. The detailed amino acid composition is listed in Table 3 and Table 4.

### 3.4. Fatty Acid Composition

The fatty acid analysis revealed the presence of various fatty acids in both muscles and guts, including capric acid, lauric acid, myristic acid, palmitic acid, stearic acid, oleic acid, linoleic acid, arachidonic acid (ARA), eicosapentaenoic acid (EPA), docosahexaenoic acid (DHA), erucic acid, omega-3 fatty acids (ω3), and omega-6 fatty acids (ω6). Caprylic acid was only observed in muscle. The thrombogenic indices (TIs), atherogenic indices (AIs), and hypocholesterolemic/hypercholesterolemic indices (h/Hs) ranged from 0.02 to 0.12, 0.01 to 0.08, and 1.58 to 3.76, respectively (Table 5).

### 3.5. Heavy Metals

Heavy metal analysis showed Pb, Hg, and As contents of 0.01, <0.01, and 0.01 ppm, respectively, while Cd was not detected in any of the tested whelk samples (Table 6). The observed heavy metal concentrations were within the acceptable ranges for seafood products specified by the Korean Food Safety Authority [22].

### 3.6. Radioactivity Analysis

The studied samples showed no radioactivity for ^134^Cs/^137^Cs (standard: 100 Bq/kg or less) [15] or for ^131^I (standard: 100 Bq/kg or less) [15] (Table 7).

### 3.7. Microbial Examination

The microbial examination of the whelk samples showed TBCs ranging from 2.01 to 2.73 log CFU/g in the gut, slightly higher than the observed range in muscles (1.97 to 2.22 log CFU/g). Notably, no *E. coli* or coliforms were detected in any of the studied whelk samples (Table 8).

## 4. Discussion

The nutritional assessment of the deep-sea whelk species *B. tenuissimum* unveiled a rich array of nutrients, positioning it favorably among shellfish worldwide. This investigation aligns with prior studies on marine mollusks highlighting their high protein levels [4,23,24] and lipid contents abundant in essential fatty acids [25,26]. Notably, the presence of essential minerals, such as Ca, Na, and Fe, further underscores their nutritional significance. Overall, this study emphasizes the nutritional significance of Korean whelk species, advocating their potential inclusion in health-conscious human diets.

Proteins play a crucial role in mollusks, functioning as an essential energy source [27]. The protein content in *B. tenuissimum* (ranging from 13.54% to 20.47%) is comparable to those of herbivorous gastropods like abalone (Haliotidae; 18.0 ± 0.7%) [28] and *Cookia sulcata* (17.5 ± 1.5%) [29]. While predatory gastropods like *Hexaplex trunculus* (48%) [30], *Rapana venosa* (55.88 ± 2.04%) [31] and *Babylonia spirata* (53.86%) [4] often exhibit higher protein contents, *B. tenuissimum*’s levels (up to 20.47%) suggest its potential as an excellent protein source. However, it is important to note that protein contents may vary depending on factors such as season, body parts, and organismal variation [32]. Beyond proteins, fats play a significant role in *B. tenuissimum*’s flesh. The lipid contents observed in *B. tenuissimum* (0.85 to 1.18% in muscles and 7.18 to 8.59% in guts) align perfectly with previous studies on gastropods reporting low lipid contents (0.5–10% *w*/*w*) [4,28,33,34,35,36]. Both the muscle and gut tissues of the studied samples showed fat contents of less than 10%, which confirms that *B. tenuissimum* flesh is suitable for inclusion in a high-protein and low-fat human diet. This study standardized comparisons by utilizing muscle and gut tissues of whelks collected from the same location and at the same time, yet future investigations into temporal and spatial changes are recommended.

Amino acid composition analysis of whelk muscles and guts revealed the presence of all 18 amino acids, with aspartic acid (up to 1.89%), glutamic acid (up to 2.42%), leucine (up to 1.45%), lysine (up to 1.41%), and arginine (up to 1.15%) contributing to the observed protein contents in *B. tenuissimum* (up to 20.47%). The free amino acids showed total amounts ranging from 8188.32 ± 29.82 to 10,680.9 ± 37.93 mg/kg, with taurine (1697.33 mg/kg), sarcosine (up to 1663.61 mg/kg), arginine (up to 1400 mg/kg), glutamic acid (up to 1017.92 mg/kg), alanine (up to 661.01 mg/kg), and aspartic acid (up to 619.50 mg/kg) being the predominant free amino acids. Although the lysine content was comparatively low when compared to certain legumes [soybean (6.40 g/100 g) [37] and cowpea (2.8 g/100 g) [38], the studied whelk meat demonstrated appreciable values of essential amino acids compared to legumes [37,38], goat and beef meat [39], as well as *Buccinum inclytum* whelk [3]. The observed contents of essential amino acids, including branched-chain amino acids (valine, leucine, and isoleucine), position *B. tenuissimum* as a potential contributor to meeting human dietary requirements.

The fatty acid composition analysis of *B. tenuissimum*’s muscle and gut tissues revealed palmitic acid (C16:0) as the dominant saturated fatty acid, consistent with studies on other mollusks like *Haliotis fulgens*, the pulmonate land snail (*Helix aspersamaxima*), the oyster (*Crassostrea rhizophorae*), and Turban snails [4,40,41,42]. The high proportion of palmitic acid may be attributed to the whelk’s primary diet of macroalgae, known for their richness in C16 saturated fatty acids [36,43]. Additionally, other fatty acids, such as stearic acid (C18:0), oleic acid (C18:1), eicosenoic acid (C20:1), eicosadienoic acid (C20:2), eicosapentaenoic acid (C20:5), docosahexaenoic acid (C22:6), and lignoceric acid (C24:0), were predominant in *B. tenuissimum*. These findings, in line with observations from other gastropods, including *Haliotis laevigata*, *Haliotis rubra*, *Turbo cornutus*, and Turban snails [4,36], indicate a similarity in fatty acid profiles among these species. Furthermore, *B. tenuissimum*’s muscles and guts were found to be rich in essential fatty acids, such as EPA, DHA, and ARA. These essential fatty acids cannot be synthesized by mollusks and must be obtained from their diets [4]. Consequently, subtle differences in the diets of different whelks could explain the variations in fatty acid composition among different-sized whelks collected from the same location.

The long-chain polyunsaturated fatty acids (PUFAs), such as docosapentaenoic acid (DPA), ARA, EPA, and DHA, are well-known for their role in lowing cholesterol levels, reducing coronary heart disease, and preventing inflammation and arteriosclerosis [44,45]. Consistent with the findings from this study, these PUFAs have been previously reported in other marine mollusks [4,43,46,47,48,49,50,51]. The ratio of *n*-3 to *n*-6 fatty acids serves as a valuable indicator of the nutritional value of fatty foods [4]. Furthermore, a maximum *n*-3 to *n*-6 ratio of 4 is recommended for the human diet [41], and an imbalance in this ratio has been associated with various diseases, including cancer [42,43]. In this study, the ratio of *n*-3 to *n*-6 in the whelks’ muscles and guts was found to be less than 1 (ranging from 0.13 to 0.34), which is considered a healthful ratio due to the high quantities of EPA, DHA, and ARA. This is in line with the previous studies on other snails, such as *Lunella undulatus* (0.90) [52], *Haliotis* spp. (1.2 to 1.70) [52], and Turban snails (<1) [4].

Additionally, the calculated AI, TI, and h/H values for whelks ranged from 0.02 to 0.12, 0.01 to 0.08, and 1.58 to 3.76, respectively. These values were lower than those observed in farmed salmon (AI: 0.19; TI: 0.22) and wild salmon (AI: 0.43; TI: 0.18) as reported by Molversmyr et al. [53]. It has been reported that AI and TI values surpassing 1.0 may have detrimental effects on human health [54,55]. Interestingly, the observed values for AI and TI fell within this range, indicating the beneficial health prospect of whelk muscles and guts. Additionally, the observed h/H values in this study (ranging from 1.58 to 3.76) fall within the range reported for other shellfish species (1.73 to 4.75) and fish (1.54 to 4.83) [56]. In comparison to the *n*-6/*n*-3 ratio, the h/H ratio may better indicate the impact of fatty acid composition on various diseases, including cardiovascular disorders [56]. The favorable ratio of *n*-3 to *n*-6 fatty acids, along with the h/H ratio, AI, and TI values and the high content of essential fatty acids, particularly EPA, DHA, and ARA, in *B. tenuissimum*, further supports its potential contribution to a healthful diet. However, it is important to note that, like the AI and TI indices, the h/H ratio also has limitations, including its incorporation of various fatty acids like MUFAs and the potential for assigning different weights to different fatty acids.

Mollusks, including *B. tenuissimum*, serve as valuable bioindicators for minerals and trace elements in the environment [57]. *B. tenuissimum* whelks exhibited high concentrations of essential macroelements, including Na, Ca, Fe, and K, making them a potential source of these essential minerals in human consumption. While Na levels were notably higher than those observed in other studies [29,58], K levels were relatively lower, ranging from 0.35 to 0.38 g/100 g. The Ca levels (ranging from 57.72 to 284.58 mg/100 g) and Fe levels (ranging from 0.87 to 6.09 mg/100 g) in the flesh of *B. tenuissimum* remained within safe limits for human consumption, which are 2.3 g per day for Ca and 45 mg per day for Fe [59].

The concentrations of heavy metals (Pb, Hg, and As) in *B. tenuissimum* were within the acceptable ranges for seafood products [22], ensuring their safety for consumption. Cd was not detected in any of the tested whelk samples. The radioactivity analysis for radioactive isotopes, such as ^131^I and ^134^Cs/^137^Cs, showed no detectable radioactivity, meeting safety standards. The TBCs in the gut (ranging from 2.01 to 2.73 log CFU/g) were slightly higher than those in the muscle (ranging from 1.97 to 2.22 log CFU/g) but remained within acceptable ranges. Notably, no *E. coli* or coliforms were detected in any of the studied whelk samples.

However, it is crucial to acknowledge the potential variability in the mineral composition of marine foods due to seasonal and biological factors, including sex, age, size, sexual maturity, and species. Additionally, factors such as provision of food, geographical location, and environmental conditions, including environmental pollution, temperature, and humidity can contribute to variations [4,57,60,61,62]. Future studies should comprehensively explore these factors to provide a more nuanced understanding of the mineral composition of marine whelks.

## 5. Conclusions

This study reports an initial comprehensive examination of the nutritional composition and safety evaluation of deep-sea whelks sourced from the Republic of Korea. *B. tenuissimum* exhibited a promising nutritional profile, being rich in proteins, essential fatty acids, and essential minerals. In addition, the safety evaluations, including heavy metal, radioactivity, and microbial content evaluations, indicate that *B. tenuissimum* is safe for human consumption. The outcomes of this study suggest minimal disparities in nutritional composition among differently sized whelks, emphasizing the potential of both muscle and gut tissues as healthful food sources. This places whelks in a category similar to other well-accepted mollusks like abalone. To deepen our understanding of the factors influencing proximate composition variations in *B. tenuissimum* whelks, further research on the effects of genetic, ecological, and abiotic factors is essential. This includes exploring the impact of age, location, environmental pollution, reproductive cycle, sex, and food availability. Considering that all samples in this study were obtained in January, seasonal effects should also be taken into consideration. Such additional investigations could contribute to fostering consumer acceptance of *B. tenuissimum* as a novel meat source. This, in turn, could add substantial value to the mollusk fishing industry, particularly in countries such as the Republic of Korea and Japan, where a limited number of species are commonly consumed by the majority of the population.

## Figures and Tables

**Table 1:** Measured physical characteristics of muscles and guts of different-sized deep-sea whelks (*B. tenuissimum* Kuroda).

Characteristic	Large-Sized Whelks	Medium-Sized Whelks	Small-Sized Whelks
Shell height (mm)	78.22 ± 4.99 ^a^	60.31 ± 6.24 ^b^	44.06 ± 3.63 ^c^
Shell breadth (mm)	47.69 ± 6.71 ^a^	34.99 ± 5.82 ^b^	28.98 ± 3.60 ^b^
Total weight (g)	55.90 ± 5.97 ^a^	31.88 ± 8.63 ^b^	14.41 ± 2.42 ^c^
Shell weight (g)	9.06 ± 2.09 ^a^	6.69 ± 1.70 ^ab^	3.87 ± 0.74 ^b^
Muscle weight (g)	20.18 ± 3.68 ^a^	12.42 ± 3.68 ^b^	5.51 ± 1.40 ^c^
Gut weight (g)	23.94 ± 4.17 ^a^	11.66 ± 5.59 ^b^	3.74 ± 0.91 ^b^

Values are means ± SDs of 25 whelks in each replication (*n* = 25). Different superscript letters (^a–c^) in each column for each characteristic indicate significant differences among means by Duncan’s test (*p* < 0.05).

**Table 2:** Proximate composition of muscles and guts of different-sized deep-sea whelks (*B. tenuissimum* Kuroda).

Component	Unit	Large-Sized Whelks		Medium-Sized Whelks		Small-Sized Whelks	
		Muscle	Gut	Muscle	Gut	Muscle	Gut
Crude protein	g/100 g	13.54 ± 0.04	16.38 ± 0.07	13.82 ± 0.05	16.75 ± 0.06	15.24 ± 0.05	20.47 ± 0.08
Carbohydrate	g/100 g	12.81 ± 0.04	2.84 ± 0.02	4.53 ± 0.02	2.48 ± 0.02	2.48 ± 0.01	1.55 ± 0.02
Sugars	g/100 g	0.80 ± 0.004	1.17 ± 0.02	1.18 ± 0.01	1.04 ± 0.03	1.10 ± 0.02	1.40 ± 0.03
Fat	g/100 g	0.94 ± 0.005	7.18 ± 0.01	0.85 ± 0.01	8.39 ± 0.04	1.18 ± 0.01	8.59 ± 0.01
Saturated fat	g/100 g	0.46 ± 0.002	1.90 ± 0.02	0.32 ± 0.01	2.14 ± 0.05	0.42 ± 0.01	2.12 ± 0.04
Trans fat	g/100 g	n.d.	n.d.	n.d.	n.d.	n.d.	n.d.
Cholesterol	mg/100 g	110.30 ± 0.54	367.30 ± 1.81	117.78 ± 0.53	366.19 ± 1.76	116.38 ± 0.52	469.57 ± 2.41
Crude ash	g/100 g	2.66 ± 0.02	2.31 ± 0.02	2.18 ± 0.03	2.15 ± 0.03	2.48 ± 0.01	2.81 ± 0.01
Energy	kJ/100 g	476.39 ± 2.32	592.04 ± 2.97	339.11 ± 1.64	637.77 ± 3.18	341.00 ± 1.72	692.00 ± 3.21
Sodium	mg/100 g	715.87 ± 3.62	572.77 ± 2.93	609.26 ± 3.14	507.63 ± 2.55	669.24 ± 3.32	482.69 ± 2.45
Calcium	mg/100 g	115.34 ± 0.64	86.96 ± 0.44	57.72 ± 0.30	76.00 ± 0.37	68.97 ± 0.33	284.58 ± 1.53
Cholecalciferol	IU/100 g	n.d.	n.d.	n.d.	n.d.	n.d.	n.d.
Potassium	g/100 g	0.37 ± 0.002	0.35 ± 0.002	0.35 ± 0.001	0.37 ± 0.002	0.38 ± 0.001	0.37 ± 0.002
Dietary fiber	g/100 g	1.25 ± 0.006	1.81 ± 0.007	1.46 ± 0.009	1.94 ± 0.010	1.33 ± 0.008	1.95 ± 0.009
Iron	mg/100 g	1.28 ± 0.004	4.11 ± 0.006	0.87 ± 0.007	5.60 ± 0.029	0.92 ± 0.005	6.09 ± 0.04

Values are means ± SDs of three replicates. n.d.: Not detected.

**Table 3:** Bound amino acid composition of muscles and guts of different-sized deep-sea whelks (*B. tenuissimum* Kuroda).

Amino Acid Content (%)	Large-Sized Whelks		Medium-Sized Whelks		Small-Sized Whelks	
	Muscle	Gut	Muscle	Gut	Muscle	Gut
Aspartic acid	1.24 ± 0.006	1.55 ± 0.008	1.21 ± 0.005	1.52 ± 0.008	1.31 ± 0.006	1.89 ± 0.009
Threonine	0.54 ± 0.007	0.68 ± 0.008	0.56 ± 0.007	0.74 ± 0.009	0.58 ± 0.006	0.87 ± 0.008
Serine	0.58 ± 0.006	0.53 ± 0.007	0.62 ± 0.006	0.68 ± 0.003	0.61 ± 0.004	0.72 ± 0.004
Glutamic acid	1.87 ± 0.009	1.99 ± 0.010	1.80 ± 0.007	1.95 ± 0.008	1.96 ± 0.014	2.42 ± 0.015
Proline	0.53 ± 0.005	0.61 ± 0.003	0.55 ± 0.002	0.62 ± 0.003	0.55 ± 0.004	0.73 ± 0.006
Glycine	0.86 ± 0.007	0.76 ± 0.004	0.86 ± 0.003	0.79 ± 0.003	0.84 ± 0.003	0.93 ± 0.003
Alanine	0.76 ± 0.004	0.84 ± 0.006	0.75 ± 0.006	0.84 ± 0.007	0.80 ± 0.006	1.00 ± 0.009
Valine	0.56 ± 0.003	0.80 ± 0.004	0.52 ± 0.004	0.73 ± 0.005	0.60 ± 0.002	0.98 ± 0.008
Isoleucine	0.45 ± 0.003	0.69 ± 0.006	0.40 ± 0.001	0.63 ± 0.002	0.48 ± 0.002	0.86 ± 0.005
Leucine	0.91 ± 0.003	1.18 ± 0.009	0.88 ± 0.004	1.13 ± 0.008	0.98 ± 0.004	1.45 ± 0.007
Tyrosine	0.29 ± 0.001	0.33 ± 0.001	0.33 ± 0.001	0.43 ± 0.002	0.31 ± 0.001	0.47 ± 0.002
Phenylalanine	0.40 ± 0.003	0.67 ± 0.004	0.40 ± 0.002	0.63 ± 0.003	0.44 ± 0.003	0.84 ± 0.005
Histidine	0.25 ± 0.001	0.44 ± 0.002	0.25 ± 0.001	0.42 ± 0.002	0.26 ± 0.001	0.51 ± 0.003
Lysine	0.82 ± 0.004	1.14 ± 0.008	0.80 ± 0.007	1.11 ± 0.010	0.89 ± 0.008	1.41 ± 0.012
Arginine	1.11 ± 0.009	0.92 ± 0.007	1.08 ± 0.006	0.94 ± 0.005	1.15 ± 0.008	1.13 ± 0.007
Cystine	0.20 ± 0.001	0.45 ± 0.002	0.20 ± 0.001	0.42 ± 0.002	0.21 ± 0.001	0.54 ± 0.004
Methionine	0.33 ± 0.002	0.52 ± 0.003	0.34 ± 0.002	0.45 ± 0.003	0.37 ± 0.002	0.57 ± 0.004
Tryptophan	0.07 ± 0.001	0.83 ± 0.004	0.07 ± 0.001	0.73 ± 0.004	0.08 ± 0.001	0.21 ± 0.001
Total amount	11.77 ± 0.05	14.93 ± 0.072	11.62 ± 0.061	14.76 ± 0.065	12.42 ± 0.052	17.53 ± 0.073

Values are means ± SDs of three replicates.

**Table 4:** Free amino acid composition of muscles and guts of different-sized deep-sea whelks (*B. tenuissimum* Kuroda).

Free Amino Acids (mg/kg)	Large-Sized Whelks		Medium-Sized Whelks		Small-Sized Whelks	
	Muscle	Gut	Muscle	Gut	Muscle	Gut
Phosphoserine	n.d.	n.d.	n.d.	n.d.	n.d.	n.d.
Taurine	1558.81 ± 7.80	1251.29 ± 6.24	1566.77 ± 7.62	1357.99 ± 6.53	1697.33 ± 8.32	1493.12 ± 6.79
Phospho ethanol amine	n.d.	n.d.	n.d.	n.d.	n.d.	n.d.
Urea	n.d.	n.d.	n.d.	n.d.	n.d.	n.d.
Aspartic acid	566.43 ± 2.77	473.23 ± 1.83	544.31 ± 2.44	478.17 ± 1.85	476.35 ± 1.87	619.50 ± 3.10
Threonine	256.70 ± 1.15	222.60 ± 1.08	254.56 ± 2.02	246.22 ± 1.89	240.12 ± 2.34	298.78 ± 2.13
Serine	373.93 ± 1.92	293.67 ± 1.83	367.94 ± 2.11	296.06 ± 1.98	347.66 ± 2.25	355.70 ± 3.03
Glutamic acid	754.11 ± 4.03	751.34 ± 4.14	741.02 ± 3.87	824.52 ± 4.19	662.21 ± 3.62	1017.92 ± 5.67
Glutamine	n.d.	n.d.	n.d.	n.d.	n.d.	n.d.
Sarcosine	1663.61 ± 8.12	1025.02 ± 5.11	1590.42 ± 7.85	166.32 ± 0.84	1514.75 ± 6.98	1269.15 ± 6.17
α-amino adipic acid	n.d.	n.d.	n.d.	n.d.	n.d.	n.d.
Hydroxy proline	130.45 ± 0.63	45.79 ± 0.25	121.91 ± 0.64	48.12 ± 0.14	69.47 ± 0.54	35.84 ± 0.08
Proline	391.85 ± 1.94	475.03 ± 1.98	351.22 ± 1.85	486.61 ± 2.34	307.14 ± 1.87	445.34 ± 2.55
Glycine	427.26 ± 3.12	363.36 ± 1.74	396.16 ± 2.52	345.61 ± 1.95	338.34 ± 2.35	391.01 ± 1.87
Alanine	628.99 ± 3.52	584.17 ± 3.13	650.31 ± 4.23	649.75 ± 3.94	597.17 ± 4.31	661.01 ± 4.16
Citrulline	n.d.	n.d.	n.d.	n.d.	n.d.	n.d.
α-amino-n-butyric acid	n.d.	n.d.	n.d.	n.d.	n.d.	n.d.
Valine	270.25 ± 1.13	278.05 ± 1.05	276.08 ± 1.24	288.25 ± 2.13	269.31 ± 2.53	371.37 ± 2.44
Cystine	n.d.	n.d.	n.d.	n.d.	n.d.	n.d.
Methionine	181.40 ± 0.94	150.57 ± 0.85	194.64 ± 0.99	143.15 ± 0.74	185.28 ± 1.04	189.87 ± 0.87
Cystathionine	n.d.	n.d.	n.d.	n.d.	n.d.	n.d.
Isoleucine	177.34 ± 0.85	204.49 ± 1.12	184.90 ± 0.89	215.66 ± 0.98	185.08 ± 0.97	284.03 ± 1.07
Leucine	383.72 ± 1.76	423.31 ± 2.13	392.92 ± 1.85	431.30 ± 2.24	385.90 ± 1.53	575.09 ± 2.38
Tyrosine	235.46 ± 1.11	277.45 ± 1.03	231.17 ± 1.14	269.24 ± 1.21	228.32 ± 1.55	336.61 ± 1.92
Phenylalanine	157.29 ± 0.74	208.36 ± 0.95	170.11 ± 0.84	214.12 ± 0.94	161.30 ± 0.73	277.88 ± 1.02
β-Alanine	n.d.	n.d.	n.d.	n.d.	n.d.	n.d.
β-Amino isobutyric acid	n.d.	n.d.	n.d.	n.d.	n.d.	n.d.
γ-Amino-n-butyric acid	2.07 ± 0.001	n.d.	3.17 ± 0.003	n.d.	2.89 ± 0.002	n.d.
Ethanol amine	27.54 ± 0.13	105.45 ± 0.53	26.25 ± 0.12	117.97 ± 0.62	35.25 ± 0.22	157.47 ± 0.86
Tryptophan	n.d.	n.d.	n.d.	n.d.	n.d.	n.d.
Ammonia	86.16 ± 0.43	121.10 ± 0.61	87.84 ± 0.45	145.83 ± 0.78	99.79 ± 0.50	170.26 ± 0.85
Hydroxylysine	n.d.	n.d.	n.d.	n.d.	n.d.	n.d.
Ornithine	23.75 ± 0.14	51.67 ± 0.23	32.31 ± 0.16	46.91 ± 0.24	102.96 ± 0.50	45.01 ± 0.28
Lysine	299.65 ± 1.45	396.54 ± 1.98	329.49 ± 1.65	427.29 ± 2.24	329.47 ± 1.65	537.61 ± 2.86
1-Methylhistidine	n.d.	n.d.	n.d.	n.d.	n.d.	n.d.
Histidine	176.67 ± 0.87	219.05 ± 1.03	196.35 ± 0.97	251.37 ± 1.24	143.15 ± 0.72	216.39 ± 1.08
3-Methylhistidine	n.d.	n.d.	n.d.	n.d.	n.d.	n.d.
Anserine	n.d.	n.d.	n.d.	n.d.	n.d.	n.d.
Carnosine	n.d.	n.d.	n.d.	n.d.	n.d.	n.d.
Arginine	1400.78 ± 7.15	828.49 ± 4.12	1314.95 ± 6.53	737.86 ± 3.75	1291.95 ± 6.44	931.94 ± 4.44
Total amount	10,174.22 ± 40.77	8750.03 ± 34.73	10,024.8 ± 40.11	8188.32 ± 29.82	9671.19 ± 37.32	10,680.9 ± 37.93

Values are means ± SDs of three replicates. n.d.: Not detected.

**Table 5:** Fatty acid composition of muscles and guts of different-sized deep-sea whelks (*B. tenuissimum* Kuroda).

Fatty Acids (g/100 g Fatty Acid)	Shorthand	Large-Sized Whelks		Medium-Sized Whelks		Small-Sized Whelks	
		Muscle	Gut	Muscle	Gut	Muscle	Gut
Caprylic acid	C8:0	0.25 ± 0.001	n.d.	0.19 ± 0.001	n.d.	0.17 ± 0.001	n.d.
Capric acid	C10:0	0.05 ± 0.000	0.01 ± 0.000	0.10 ± 0.001	0.01 ± 0.000	0.10 ± 0.001	0.02 ± 0.000
Lauric acid	C12:0	0.80 ± 0.002	0.02 ± 0.000	0.06 ± 0.000	0.02 ± 0.000	0.07 ± 0.000	0.03 ± 0.000
Myristic acid	C14:0	3.47 ± 0.017	3.09 ± 0.016	2.90 ± 0.014	3.09 ± 0.014	2.36 ± 0.012	3.11 ± 0.013
Pentadecanoic acid	C15:0	0.51 ± 0.003	0.56 ± 0.003	0.36 ± 0.002	0.48 ± 0.003	0.33 ± 0.001	0.47 ± 0.002
Palmitic acid	C16:0	20.28 ± 0.110	15.25 ± 0.074	12.11 ± 0.065	14.83 ± 0.074	11.52 ± 0.054	14.03 ± 0.065
Magaric acid	C17:0	1.43 ± 0.006	0.73 ± 0.004	1.50 ± 0.007	0.58 ± 0.003	0.14 ± 0.001	0.68 ± 0.002
Stearic acid	C18:0	17.15 ± 0.101	4.21 ± 0.015	13.44 ± 0.044	3.72 ± 0.013	14.58 ± 0.053	3.91 ± 0.013
Arachidic acid	C20:0	0.17 ± 0.001	0.10 ± 0.000	0.06 ± 0.000	0.09 ± 0.000	0.06 ± 0.000	0.08 ± 0.000
Lignoceric acid	C24:0	4.61 ± 0.012	2.54 ± 0.008	6.41 ± 0.014	2.73 ± 0.095	6.20 ± 0.021	2.35 ± 0.004
Myristoleic acid	C14:1	0.20 ± 0.001	0.11 ± 0.000	0.08 ± 0.000	0.13 ± 0.000	0.09 ± 0.000	0.12 ± 0.000
Pentadecenoic acid	C15:1	0.13 ± 0.000	0.14 ± 0.001	0.05 ± 0.000	0.14 ± 0.001	0.05 ± 0.000	0.12 ± 0.000
Palmitoleic acid	C16:1	2.20 ± 0.013	3.63 ± 0.014	1.34 ± 0.008	4.05 ± 0.025	0.73 ± 0.001	2.76 ± 0.004
Magaoleic acid	C17:1	0.37 ± 0.001	0.51 ± 0.002	0.08 ± 0.000	0.41 ± 0.002	0.18 ± 0.001	0.34 ± 0.002
Oleic acid	C18:1 *n*-9	17.17 ± 0.104	23.39 ± 0.124	9.45 ± 0.042	25.82 ± 0.135	8.27 ± 0.035	20.90 ± 0.113
Linoleic acid	C18:2 *n*-6	0.75 ± 0.003	1.30 ± 0.005	0.71 ± 0.003	1.25 ± 0.006	0.52 ± 0.002	1.11 ± 0.006
γ-Linolenic acid	C18:3 *n*-6	0.20 ± 0.001	0.21 ± 0.001	0.10 ± 0.000	0.23 ± 0.001	0.08 ± 0.000	0.13 ± 0.001
Linolenic acid	C18:3 *n*-3	0.24 ± 0.001	0.90 ± 0.002	0.20 ± 0.001	0.92 ± 0.003	0.12 ± 0.001	0.74 ± 0.004
Eicosenoic acid	C20:1 *n*-9	5.04 ± 0.024	6.82 ± 0.013	4.54 ± 0.014	7.00 ± 0.025	5.00 ± 0.013	8.60 ± 0.053
Eicosadienoic acid	C20:2 *n*-6	4.66 ± 0.012	1.68 ± 0.008	5.39 ± 0.025	1.59 ± 0.004	5.16 ± 0.014	1.71 ± 0.005
Dihomoδ-Linoleicacid	C20:3 *n*-6	0.06 ± 0.000	0.44 ± 0.001	0.10 ± 0.001	0.60 ± 0.002	0.09 ± 0.000	0.16 ± 0.001
Eicosatrienoicacid	C20:3 *n*-3	0.10 ± 0.000	0.65 ± 0.002	0.73 ± 0.003	0.65 ± 0.002	0.71 ± 0.004	0.98 ± 0.005
Arachidonic acid	C20:4 *n*-6	4.19 ± 0.015	2.89 ± 0.011	6.56 ± 0.024	2.63 ± 0.014	6.16 ± 0.025	2.91 ± 0.013
EPA	C20:5 *n*-3	9.69 ± 0.047	11.33 ± 0.053	23.73 ± 0.15	10.60 ± 0.05	26.73 ± 0.15	12.72 ± 0.06
Erucic acid	C22:1 *n*-9	0.97 ± 0.005	1.92 ± 0.007	0.35 ± 0.002	1.91 ± 0.012	0.34 ± 0.001	2.09 ± 0.013
DHA	C22:6 *n*-3	5.30 ± 0.02	17.58 ± 0.06	9.46 ± 0.046	16.54 ± 0.040	10.24 ± 0.05	19.92 ± 0.15
ΣSAFAs		48.72 ± 0.24	26.5 ± 0.13	37.13 ± 0.14	25.55 ± 0.13	35.53 ± 0.17	24.68 ± 0.13
ΣMUFAs		26.08 ± 0.15	36.52 ± 0.14	15.89 ± 0.12	39.46 ± 0.10	14.66 ± 0.08	34.93 ± 0.09
ΣPUFAs		25.19 ± 0.12	36.98 ± 0.13	46.98 ± 0.13	35.01 ± 0.12	49.81 ± 0.12	40.38 ± 0.15
Σω3	*n*-3	15.33 ± 0.09	30.46 ± 0.08	34.12 ± 0.07	28.71 ± 0.06	37.8 ± 0.11	34.36 ± 0.13
Σω6	*n*-6	5.2 ± 0.025	4.84 ± 0.024	7.47 ± 0.034	4.71 ± 0.025	6.85 ± 0.023	4.31 ± 0.014
ω6/ω3	*n*-6/*n*-3	0.34 ± 0.0002	0.16 ± 0.0001	0.22 ± 0.0001	0.16 ± 0.0001	0.18 ± 0.0001	0.13 ± 0.0001
TI		0.12 ± 0.0001	0.04 ± 0.000	0.07 ± 0.001	0.03 ± 0.000	0.02 ± 0.000	0.03 ± 0.000
AI		0.08 ± 0.001	0.01 ± 0.0000	0.01 ± 0.000	0.01 ± 0.000	0.01 ± 0.000	0.01 ± 0.000
h/H		1.58 ± 0.008	3.16 ± 0.016	3.35 ± 0.017	3.27 ± 0.016	3.76 ± 0.019	3.42 ± 0.016

Values are means ± SDs of three replicates. n.d.: Not detected; EPA: Eicosapentaenoic acid; DHA: Docosahexaenoic acid; ΣSAFAs: Total saturated fatty acids; ΣMUFAs: Total monounsaturated fatty acids; ΣPUFAs: Total polyunsaturated fatty acids; Σω3: Total omega-3 fatty acids; Σω6: Total omega-6 fatty acids; ω6/ω3: Omega-6 to omega-3 fatty acid ratio; TI: Thrombogenic index; AI: Atherogenic index; h/H: Hypocholesterolemic/hypercholesterolemic index.

**Table 6:** Heavy metals detected in muscles and guts of different-sized deep-sea whelks (*B. tenuissimum* Kuroda).

Heavy Metal Content (ppm)	Large-Sized Whelks		Medium-Sized Whelks		Small-Sized Whelks		Standard *
	Muscle	Gut	Muscle	Gut	Muscle	Gut	
Pb	0.01	0.01	0.01	0.01	0.01	0.01	0.5
Cd	n.d.	n.d.	n.d.	n.d.	n.d.	n.d.	0.5
Hg	<0.01	<0.01	<0.01	<0.01	<0.01	<0.01	0.5
As	0.01	0.01	0.01	0.01	0.01	0.01	0.1

Pb: Lead; Cd: Cadmium; Hg: Mercury; As: Arsenic; n.d.: Not detected. * Heavy metal standards for seafood products according to the Korean Food Safety Authority [22].

**Table 7:** Radioactivity analysis of muscles and guts of different-sized deep-sea whelks (*B. tenuissimum* Kuroda).

Radioisotope (Bq/kg)	Large-Sized Whelks		Medium-Sized Whelks		Small-Sized Whelks	
	Muscle	Gut	Muscle	Gut	Muscle	Gut
^131^I	n.d.	n.d.	n.d.	n.d.	n.d.	n.d.
^134^Cs/^137^Cs	n.d.	n.d.	n.d.	n.d.	n.d.	n.d.

^131^I: Iodine 131 radioactive isotope; ^134^Cs/^137^Cs: Cesium 134/137 radioactive isotope; n.d.: Not detected.

**Table 8:** Pathogenic microorganisms in muscles and guts of different-sized deep-sea whelks (*B. tenuissimum* Kuroda).

Pathogenic Microorganism (log CFU/g)	Large-Sized Whelks		Medium-Sized Whelks		Small-Sized Whelks	
	Muscle	Gut	Muscle	Gut	Muscle	Gut
TBC	2.09 ± 0.12 ^b^	2.21 ± 0.20 ^b^	2.49 ± 0.27 ^ab^	2.51 ± 0.46 ^ab^	2.71 ± 0.52 ^ab^	2.92 ± 0.19 ^a^
*Escherichia coli*	n.d.	n.d.	n.d.	n.d.	n.d.	n.d.
Coliforms	n.d.	n.d.	n.d.	n.d.	n.d.	n.d.

Values are means ± SDs of 25 whelks in each replication (*n* = 25). TBC: Total bacterial count; n.d.: Not detected. Different superscript letters (^a,b^) in each column for each characteristic indicate significant differences among means by Duncan’s test (*p* < 0.05).

## Data Availability

The original contributions presented in the study are included in the article/Supplementary Material; further inquiries can be directed to the corresponding authors.

## Supplementary Materials

The following supporting information can be downloaded at: https://www.mdpi.com/article/10.3390/foods13081169/s1, Table S1: Analytical conditions of the ICP spectrometer used for heavy metal analysis.
