# Supplementary material for: Nutritional Composition and Safety Aspects of Deep-Sea Whelks (Buccinum tenuissimum Kuroda)
Source: Foods. 2024 Apr 11;13(8):1169. doi: 10.3390/foods13081169 (PMC11049162; doi:10.3390/foods13081169)
Supplement: Supplementary file 1 [file foods-13-01169-s001.zip › foods-2932889-supplementary.pdf]

foods

MDPI

Article

Nutritional Composition and Safety Aspects of Deep-Sea Whelks (*Buccinum tenuissimum* Kuroda)

Sana Mansoor [1,†], Jin-Hwa Lee [1,†], Khawaja Muhammad Imran Bashir [1,2], Jae-Hak Sohn [3,*] and Jae-Suk Choi [1,*]

[1] Department of Seafood Science and Technology, Institute of Marine Industry, Gyeongsang National University, Tongyeong 53064, Republic of Korea; sanamansoorahmad@gmail.com (S.M.); evolution_5237@gnu.ac.kr (J.-H.L.); imranbashir@gnu.ac.kr (K.M.I.B.)
[2] German Engineering Research and Development Center for Life Science Technologies in Medicine and Environment, Busan 46742, Republic of Korea
[3] Department of Food Science and Culinary Arts, College of Health and Welfare, Silla University, Busan 46958, Republic of Korea
* Correspondence: jhsohn@silla.ac.kr (J.-H.S.); jsc1008@gnu.ac.kr (J.-S.C.); Tel.: +82-51-999-5629 (J.-H.S.); +82-55-772-9142 (J.-S.C.)
† These authors contributed equally to this work.

Table S1. Analytical conditions of ICP spectrometer used for heavy metal analysis.

Parameter	Condition
RF power	1350 W
Nebulizer flush pump rate (rpm)	100
Nebulizer analysis pump rate (rpm)	100
Nebulizer pump relaxation time	5 sec
Flush time	30 sec
Rinse time	10 sec
Stabilization time	30 sec
Nebulizer flow	0.61 L/min
Auxiliary gas	1.00 L/min
Light source	ICAP
Pump tubing type	tygon-orange

ICP: Inductively coupled plasma; RF: Radio frequency; ICAP: White LED light.
